# Supplementary figures and images for: Green synthesized silver nanoparticles from Moringa: Potential for preventative treatment of SARS-CoV-2 contaminated water
Source: PLoS One. 2025 Dec 22;20(12):e0338800. doi: 10.1371/journal.pone.0338800 (PMC12721540; doi:10.1371/journal.pone.0338800)

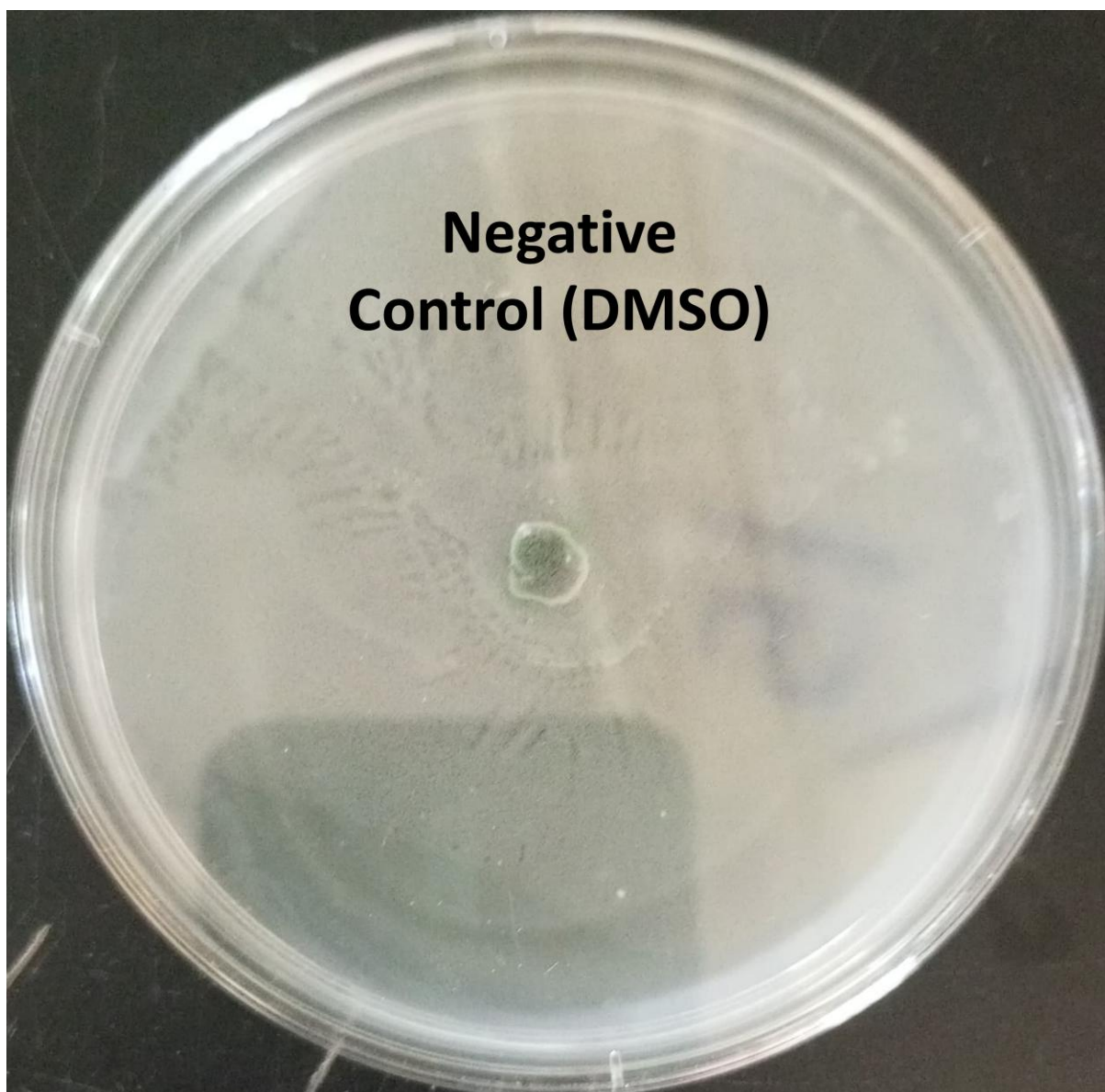

**S1 Figure. Showing negative control (NC) containing DMSO without the AgNPmo**

Supplement: S1 Fig — (PDF) [file pone.0338800.s001.pdf]
