# Supplementary material for: Green synthesized silver nanoparticles from Moringa: Potential for preventative treatment of SARS-CoV-2 contaminated water
Source: PLoS One. 2025 Dec 22;20(12):e0338800. doi: 10.1371/journal.pone.0338800 (PMC12721540; doi:10.1371/journal.pone.0338800)
Supplement: S2 Fig — (PDF) [file pone.0338800.s002.pdf]

(a) (b) (c)

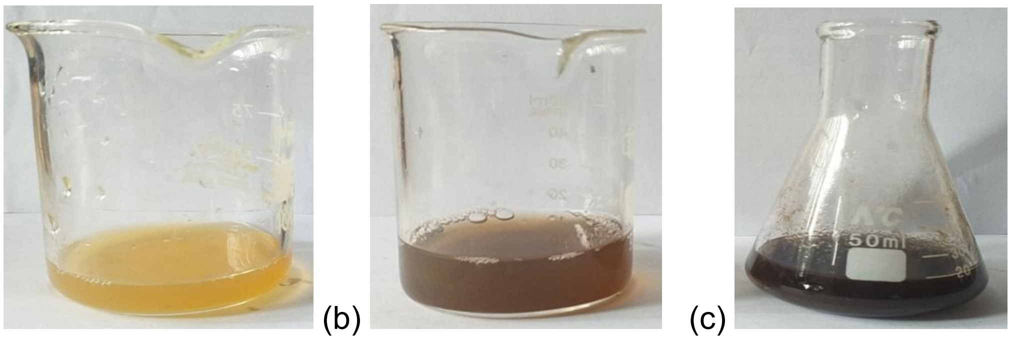

S2 Figure. **Visual observation of AgNPmo synthesis showing the progressive color change of the reaction mixture** (a) 0 min – yellowish prior to nanoparticle formation, (b) 5 min – brown indicating onset of nanoparticle formation, and (c) 10 min – reddish brown brown corresponding to complete AgNP formation.
