# Supplementary material for: Green synthesized silver nanoparticles from Moringa: Potential for preventative treatment of SARS-CoV-2 contaminated water
Source: PLoS One. 2025 Dec 22;20(12):e0338800. doi: 10.1371/journal.pone.0338800 (PMC12721540; doi:10.1371/journal.pone.0338800)
Supplement: S1 Table — (PDF) [file pone.0338800.s003.pdf]

S1. Spectrophotometry readings

Absorbance	Red Spectrum
350	0.5805
360	0.579
370	0.575
380	0.57
390	0.567
400	0.563
410	0.563
420	0.586
430	0.582
440	0.5745
450	0.5665
460	0.5525
470	0.548
480	0.545
490	0.5425
500	0.542
