# Supplementary material for: Green synthesized silver nanoparticles from Moringa: Potential for preventative treatment of SARS-CoV-2 contaminated water
Source: PLoS One. 2025 Dec 22;20(12):e0338800. doi: 10.1371/journal.pone.0338800 (PMC12721540; doi:10.1371/journal.pone.0338800)
Supplement: S2 Table — (PDF) [file pone.0338800.s004.pdf]

**S2 Table. Spectrophotometry readings**

| Absorbance | Blue Spectrum |
|---|---|
| 350 | 0.233 |
| 360 | 0.235 |
| 370 | 0.237 |
| 380 | 0.24 |
| 390 | 0.242 |
| 400 | 0.246 |
| 410 | 0.247 |
| 420 | 0.244 |
| 430 | 0.238 |
| 440 | 0.231 |
| 450 | 0.222 |
| 460 | 0.214 |
| 470 | 0.201 |
| 480 | 0.192 |
| 490 | 0.181 |
| 500 | 0.17 |
