# Supplementary material for: Green synthesized silver nanoparticles from Moringa: Potential for preventative treatment of SARS-CoV-2 contaminated water
Source: PLoS One. 2025 Dec 22;20(12):e0338800. doi: 10.1371/journal.pone.0338800 (PMC12721540; doi:10.1371/journal.pone.0338800)
Supplement: S3 Table — (PDF) [file pone.0338800.s005.pdf]

S3 Table. Values for the cytotoxicity assay

Concentration (µg/µl)	Value 1	Value 2	Mean	Standard Deviation
0	100.00	100.00	100.00	0.00
4.88	67.42	179.75	123.59	79.43
9.77	71.29	170.45	120.87	70.12
19.53	91.48	171.05	131.27	56.27
39.06	84.16	162.14	123.15	55.14
78.13	51.07	83.83	67.45	23.16
156.25	12.52	-0.76	5.88	9.39
312.5	0.71	-1.95	-0.62	1.88
625	-1.41	-1.16	-1.28	0.17
1250	-3.28	-1.25	-2.27	1.43
2500	-2.87	0.46	-1.21	2.35
5000	-4.29	2.65	-0.82	4.91
