# Supplementary material for: Green synthesized silver nanoparticles from Moringa: Potential for preventative treatment of SARS-CoV-2 contaminated water
Source: PLoS One. 2025 Dec 22;20(12):e0338800. doi: 10.1371/journal.pone.0338800 (PMC12721540; doi:10.1371/journal.pone.0338800)
Supplement: S4 Table — (PDF) [file pone.0338800.s006.pdf]

S4 Table. Zone of Inhibition (ZOI) of *P. aeruginosa* at different concentrations of the AgNPmo

Concentration (%)	Value 1	Value 2	Mean	Standard Error
100	15	16	15.5	0.5
50	13	9	11	2
25	7	8	7.5	0.5
12.5	7	6	6.5	1.5
