# Supplementary material for: Green synthesized silver nanoparticles from Moringa: Potential for preventative treatment of SARS-CoV-2 contaminated water
Source: PLoS One. 2025 Dec 22;20(12):e0338800. doi: 10.1371/journal.pone.0338800 (PMC12721540; doi:10.1371/journal.pone.0338800)
Supplement: S5 Table — (PDF) [file pone.0338800.s007.pdf]

S5 Table. Zone of Inhibition (ZOI) of *S. aureus*at different concentrations of the AgNPmo

Concentration (%)	Value 1	Value 2	Mean	Standard Error
100	9	5	7	2
50	4	4	4	0
25	4	3	3.5	0.5
12.5	0	0	0	0
