# Supplementary material for: Green synthesized silver nanoparticles from Moringa: Potential for preventative treatment of SARS-CoV-2 contaminated water
Source: PLoS One. 2025 Dec 22;20(12):e0338800. doi: 10.1371/journal.pone.0338800 (PMC12721540; doi:10.1371/journal.pone.0338800)
Supplement: S6 Table — (PDF) [file pone.0338800.s008.pdf]

S6 Table. Inhibitory effect of the biosynthesized nanoparticles against SARS-CoV-2 N-gene

	0HR				24hrs				48HRS			
Concentration (µg/µl)	Value 1	Value 2	Mean	Standard Error	Value 1	Value 2	Mean	Standard Error	Value 1	Value 2	Mean	Standard Error
38 µg/µl	23.01	23.18	23.10	0.085	25.36	24.26	24.81	0.55	27.02	26.69	26.86	0.165
19 µg/µl	24.11	24.06	24.08	0.035	24.85	24.64	24.74	0.105	27.46	25.91	26.68	0.775
9.5 µg/µl	24.59	24.49	24.54	0.05	25.61	24.19	24.90	0.71	28.03	26.54	27.29	0.745
4.25 µg/µl	23.81	24.27	24.04	0.23	24.39	23.67	24.03	0.36	24.39	26.84	25.62	1.225
SC	22.43	22.83	22.63	0.2	23.55	23.79	23.67	0.12	23.68	23.66	23.67	0.105
