# Supplementary material for: Green synthesized silver nanoparticles from Moringa: Potential for preventative treatment of SARS-CoV-2 contaminated water
Source: PLoS One. 2025 Dec 22;20(12):e0338800. doi: 10.1371/journal.pone.0338800 (PMC12721540; doi:10.1371/journal.pone.0338800)
Supplement: S7 Table — (PDF) [file pone.0338800.s009.pdf]

S7 Table. Inhibitory effect of the biosynthesized nanoparticles against SARS-CoV-2 ORF-Lab 1 gene

	0HR				24hrs				48HRS			
Concentration (µg/µl)	Value 1	Value 2	Mean	Standard Error	Value 1	Value 2	Mean	Standard Error	Value 1	Value 2	Mean	Standard Error
38 µg/µl	23.073	23.315	23.194	0.121	26.17	25.659	25.9145	0.2555	27.878	26.36	27.119	0.759
19 µg/µl	24.175	23.397	23.786	0.389	25.636	25.381	25.5085	0.1275	28.328	25.603	26.9655	1.3625
9.5 µg/µl	25.745	24.487	25.116	0.629	26.046	25.105	25.5755	0.4705	28.936	27.589	28.2625	0.6735
4.25 µg/µl	24.059	24.05	24.0545	0.0045	25.344	23.555	24.4495	0.8945	28.973	27.571	28.272	0.701
SC	23.973	23.731	23.852	0.221	23.91	24.245	24.0775	0.2675	24.352	24.051	24.2015	0.1505
