# Supplementary material for: Green synthesized silver nanoparticles from Moringa: Potential for preventative treatment of SARS-CoV-2 contaminated water
Source: PLoS One. 2025 Dec 22;20(12):e0338800. doi: 10.1371/journal.pone.0338800 (PMC12721540; doi:10.1371/journal.pone.0338800)
Supplement: S9 Table — (PDF) [file pone.0338800.s011.pdf]

S9 Table. Major absorption features of AgNPmo identified from UV–Vis spectrophotometry

Wavelength (nm)	Peak Assignment	Functional group
420	AgNP surface plasmon resonance (SPR)	Silver nanoparticle formation
455	Minor absorption shoulder	Phenolic compound from *Moringa* extract
480	Broad absorption tail	Conjugated compounds in extract
