# Supplementary material for: Green synthesized silver nanoparticles from Moringa: Potential for preventative treatment of SARS-CoV-2 contaminated water
Source: PLoS One. 2025 Dec 22;20(12):e0338800. doi: 10.1371/journal.pone.0338800 (PMC12721540; doi:10.1371/journal.pone.0338800)
Supplement: Attachment — Submitted filename: Response to Reviewers.docx [file pone.0338800.s013.docx]

**Reviewer #1:**

**Comment:** The study is equally contributed in antibacterial activity, so re-consider to change the title accordingly.
Moringa oleifera leaf extracts possess biocompounds with antioxidant and antibacterial activities (24).: Better include the major bioactive compounds in M.oleifera leaf extracts.

**Response:** We appreciate your comment. The focus of this study is to show the antiviral activity of the AgNPmo. However, we first tested AgNPmo on bacterial isolates to affirm its effect on biological cells. Therefore, changing the topic to reflect the antibacterial activities of AgNPmo would be misleading, as that is not the main focus or conclusion of our study.

**Comment:** Quantitative RT-PCR Assay for antiviral activity: Primer details missing

**Response:** The manufacturer did not provide the primer details for the kit. The kit was validated and approved by the Centre for Human Virology and Genomics (CHVG), Nigerian Institute of Medical Research, using the Foundation for Innovative New Diagnostics (FIND) protocol, adapted from the WHO assay validation protocol. We have provided the reference to the protocol in the manuscript (Shaibu et al. 2023).

Fig 1: Explain the small insert in caption. If possible provide spectra of extracts and AgNPs alone as control.

**Response:** The small insert shows that AgNPmo has a peak of 410nm after 12 months of storage, similar to the peak shown when it was freshly prepared, suggesting that AgNPmo retains its absorbance properties over a long period of time. This has been explained in the Results and Discussion heading, under the subheading “UV-visible analysis of the AgNPmo”

Fig 2.: Very poor resolution. Image is not clear.

**Response:** We apologize for the low resolution. This was what was obtainable by the time the sample was tested. However, the red-circled parts show that the non-agglomerated particles are within the nanoscale range.

Fig 3.: Provide the major components in table or list in caption.

**Response:** The major components of the image have been provided in the caption.

Fig 4.: Provide the image for NC. Also, need to include positive control using commercial antibiotics as reference.

**Response:** We have provided the image for the negative control, which contained only the DMSO, and no zone of inhibition is visible.

Fig 5.: Did you test the cytotoxicity of leaf extracts?

**Response:** We did not test the cytotoxicity of leaf extracts separately. This is because our work focused on the effect of the silver nanoparticles and not the plant extract itself.

Fig 6.: Do not provide Ct value, its not actual expression of genes, Change to "Relative expression to HKG or percentage expression"
FIg.6: should investigate major genes such as ACE2 (Angiotensin-Converting Enzyme 2), TMPRSS2 (Transmembrane Serine Protease 2), etc....

**Response:** We thank the reviewer for this comment and input. However, we prefer to use Ct values because they provide a direct indication of the detection of the target genes' presence. We used this because it directly indicates the presence of the SARS-CoV-2 virus in water samples and monitors the concentration of the viral load, as is usually done for patients with COVID-19.

Also, our study focuses on the target genes ORF1ab and N gene, which are the gold standard for the detection of the virus.

**Comment:** The discussion should include a more in-depth comparison of the findings with previous studies, supported by appropriate references to earlier works. Currently, the discussion is concise and would benefit from elaboration and citations to strengthen its context and relevance.

**Response:** The discussions has been updated with relevant citations

**Reviewer #2:** 
Thank you for giving me the opportunity to review this manuscript. Thanks to the valuable efforts of the authors. This article is very well-organized. However, my comments on this manuscript are as follows.

**Comment:** The introduction provides a good background but lacks depth in reviewing recent literature. Incorporate more recent studies to contextualize the research within the current state of knowledge.

**Response:** We thank the reviewer for the valuable comments. The introduction section has been updated with relevant citations.

**Comment:** The method requires more precise details. Please use the reference below in the method section. <https://www.scopus.com/record/display.uri?eid=2-s2.0-85148103559&origin=inward&txGid=beff2bfbdbe858b60a401178ebbf0d89>

**Response:** the reference (Barjoee et al., 2020) has been cited in the method section.

**Comment:** Please summarize the discussion section by presenting key findings and comparisons with recent studies (last 3 years).

**Response:** The discussion section has been summarized, and the latest references included.

**Comment:** Please explain what are the limitations of this study? The discussion currently does not address the limitations of the study. Including a section that outlines any potential biases, methodological constraints, or external factors that may have influenced the results is essential for transparency and helps contextualize the findings.

**Response:** The discussion section now includes the limitations of the study.

**Comment:** It would be beneficial to suggest specific areas for future research based on the findings and limitations of this study. This can guide subsequent studies and contribute to the ongoing discourse.

**Response:** The discussion section now includes the future direction of the study.
