# Supplementary material for: Green synthesized silver nanoparticles from Moringa: Potential for preventative treatment of SARS-CoV-2 contaminated water
Source: PLoS One. 2025 Dec 22;20(12):e0338800. doi: 10.1371/journal.pone.0338800 (PMC12721540; doi:10.1371/journal.pone.0338800)
Supplement: Attachment — Submitted filename: Response to Reviewers comment.docx [file pone.0338800.s014.docx]

Response to the Reviewer

Reviewer #1: Comments:

Comment:

the antiviral activity of the AgNPmo on the SARS-CoV-2 virus: provide the evidence how did the authors claim this effect in abstract.

Response

We have revised the Abstract and Results sections to explicitly state the evidence of antiviral activity. Specifically, we report the increase in Ct values for ORF1ab and N genes in a time- and dose-dependent manner, with the strongest inhibition observed at 48 h.

Comment:

its efficacy against the bacterial isolates: How it was demonstrated by this study? provide the results, how it was confirmed.

Response:

In the report section, the inhibition zone values reported for *P. aeruginosa* (6.5–15.5 mm) and *S. aureus* (0–7 mm) demonstrate concentration-dependent antibacterial activity. Please see the result under the heading “Analysis of antimicrobial activity.”

Comment :

AgNPmo also showed low toxicity on the Vero cells: Evidence.

Response:

Evidence of low toxicity is provided in the revised manuscript. The CCK-8 cytotoxicity assay on Vero cells demonstrated an IC50 of **38 µg/ml** (Results, Fig. 5), indicating relatively low cytotoxicity at effective concentrations. Cell viability remained above 70% at doses below 100 µg/ml, with marked cytotoxicity observed only at much higher concentrations (>312.5 µg/ml). This supports the statement that AgNPmo exhibits low toxicity within the effective therapeutic range.

Comment :

The IC50 from the cytotoxicity assay demonstrated : Provide IC50 value.

Response: We have now included the IC50 value (38 µg/ml) in both the Results and Abstract, and corrected the unit discrepancy (µg/ml instead of µg/µl).

Comment :

synthesized was cost-effective: What’s the proof compared to commercial one?

Response:

We have revised the Discussion to clarify the basis of our statement. Specifically, our green synthesis approach avoids hazardous and costly reducing/stabilizing chemicals required in conventional methods, thereby lowering overall costs. To strengthen this point, we have now cited published literature reporting that biosynthesized AgNPs can cost approximately **£8 per unit**, compared to **£28 for equivalent commercial nanoparticles (** Rocha V, Ferreira-Santos P, Aguiar C, et al. Valorization of plant by-products in the biosynthesis of silver nanoparticles with antimicrobial and catalytic properties. Environ Sci Pollut Res. 2024;31:14191-14207. doi:10.1007/s11356-024-32180-w.)

Our method, which uses inexpensive *Moringa oleifera* leaves combined with microwave synthesis, is consistent with this observation and supports the economic advantage of plant-mediated AgNP synthesis (Discussion).

Comment :

These findings suggest that the nanoparticles could be a promising alternative: So the abstract concludes specifically on NPs not moringa, should describe the benefits of moringa and NPs in a distinct way for combating SARS-CoV-2.
Abstract needs to revise in a proper manner to describe the novel results findings of this study.

Response:

The Abstract has been revised to clearly distinguish the role of *Moringa oleifera* phytochemicals (as reducing/capping agents and potential bioactive contributors) from the effects of AgNPs.

Comment :

AgNPmo has the potential to be used for purification or treatment of SARS-CoV-2 contaminated water without exhibiting toxicity: this study did not investigate the objective directly in SARS-CoV-2 contaminated waste-water treatment. How does (in which way) the AgNPmo use in real water treatment.

Response:

We agree and have clarified in the Abstract and Discussion that this study did not directly test wastewater treatment. We now frame the statement cautiously as a future application potential rather than a direct claim.

Comment :

The silver nanoparticles (AgNPmo) were synthesized ...X-ray spectroscopy (EDX), and X-ray diffraction....using qPCR assay in a dose and time dependent manner. : can be deleted this general statement as already explained before.

Response:

This redundant statement has been deleted from the manuscript.

Comment :
colour change of the reaction mixture, changing from yellowish to dark brown within 5 minutes. : It’s better to provide some images to visualize to the readers.

Response: Images have been added to the supplementary document (supplementary Fig. S2).

Comment
CCK-8 assay revealed an IC50 value of 38 µg/µl for the 10mM stock concentration: But in Fig.5, the value was mentioned as "µg/ml" not µg/µl. Check the unit.

Response:

We have corrected the unit to µg/ml throughout the manuscript.

Comment :

Analysis of antimicrobial activity, Cytotoxicity assay , Quantitative RT-PCR Analysis: In all these experiments, the authors need to explain why AgNPmo showed beneficial effect? What was the major active compound from Moringa aqueous leaf extract involved? How combining AgNP with moringa affects these activities? Their major signaling pathways.

Response:

We have added a new paragraph in the Discussion explaining that phytochemicals (flavonoids and phenolics) from *Moringa* act as reducing/capping agents, stabilize AgNPs, and may contribute synergistic antimicrobial activity.

Comment :
until reddish-brown coloration was observed: It’s interesting to see this in picture.

Response: Images have been added to the supplementary document (supplementary Fig. S2).

Comment :
The Cell viability test of the synthesized AgNPmo was analyzed through a Cell Counting Kit-8 (CCK-8) on Vero cells.: Why not with SARS-CoV-2 ?

Response: We have clarified in the Discussion that Vero cells are a well-established preliminary model for nanoparticle cytotoxicity screening. Direct assays on SARS-CoV-2-infected cells require BSL-3 facilities, which were beyond the scope of this study.

Comment :
Statistical analysis : Its advised to use some reliable statistical analysis tool to conduct proper statistical significance, using excel 2016 version is not reliable.

Response:

We have re-analyzed the data using GraphPad Prism (v9). One-way ANOVA and Student’s t-test were applied as appropriate, with p-values < 0.05 considered significant. The Statistical Analysis section has been revised accordingly.

Comment :
Fig 1. Spectrophotometry analysis of the biosynthesized silver nanoparticles Fourier transforms infrared spectroscopy (FTIR) : Why the spectra was observed from 350 to 500 nm, however the author stated in method: AgNPmo nanoparticles within the spectral range of 3500 to 500 cm-1.?

Response:

This was a labeling/terminology confusion in the original draft. We have clarified the manuscript so that **Fig. 1** is explicitly described as the **UV–Vis** spectrum (350–500 nm) showing the AgNP surface plasmon resonance at **410 nm**, while **FTIR** data (3500–500 cm⁻¹) are now presented separately as **Fig. 2** with detailed peak assignments in Table 1 (see Methods: Physicochemical Characterization). These changes remove the previous ambiguity. (Manuscript: Methods- Physicochemical Characterization; Fig. 1 legend; Fig. 2 legend; Table 1).

Comment :

What was the small insert?

Response:

The insert in Fig. 1 represents the UV–Vis absorption spectrum of AgNPmo recorded 12 months after synthesis, shown alongside the spectrum recorded immediately after synthesis. This comparison illustrates the long-term stability of the nanoparticles, as the characteristic surface plasmon resonance peak at 410 nm was retained with minimal shift after storage. We have clarified this in the figure legend. (Manuscript: Fig. 1 legend, Results section).

Comment :

Mark the major peaks in spectra. Did the spectra done for major active compound from Moringa aqueous leaf extract?

Response:

In the revised manuscript, the major surface plasmon resonance peak at 410 nm has been highlighted in Fig. 1 (UV–Vis spectrum), and additional spectral features are listed in Supplementary Table S1. For the FTIR spectrum (Fig. 2), we have provided detailed peak positions and functional group assignments in Table 1. These clarifications ensure that the key spectral peaks are now clearly reported. (Manuscript: Fig. 1 legend, Fig. 2 legend, Table 1, Supplementary Table S1).

Comment:

Fig 2.: Scanning Electron Microscopy showing the particle size: The images showed just round red circle, not exact particle size. It’s advised to conduct XRD spectra combined with SEM to know the NP size and distribution.

Fig 3.: Authors should provide the particle size and distribution from XRD spectra not just diffractogram peaks corresponds to peaks crystallographic planes. Label Y axis.At present, XRD analysis of the AgNPmo reported only diffractogram peaks at crystallographic planes (27.91°, 32.37°, and 38.30°. 44.41°, 46.20°), but not NP size and distribution.
Here how did the authors make sure that the red circle is the one corresponding to the NPs?

Response: We thank the reviewer for this valuable suggestion. The Y-axis in Fig. 3 (now Fig 4.) has been labeled as “Intensity (a.u.)” in the revised figure. The XRD diffractogram confirmed the crystalline nature of AgNPmo, with characteristic peaks at 27.91°, 32.37°, 38.30°, 44.41°, and 46.20°. We acknowledge that particle size distribution could not be determined from the present data, as full-width half maximum (FWHM) values were not recorded. This limitation is now noted in the Discussion, and we propose to address it in future studies using TEM and DLS analyses.

Fig 4.: Negative control (NC) and positive control (PC) were missing in Fig 4 A and B. In Fig C and D: explain what NC in legends was and PC is missing in C and D. Provide statistical significance of test groups compared to controls (P value) in all graphs.

Response: Thank you for the observation. The legend of Fig. 4 (now figure 5) has been revised to clearly define NC and DMSO. The results are represented descriptively.

Comment:

Fig 5.: How many days the cells were cultured, mentioned in legends. The findings were contradictory to the claim by authors in abstract "AgNPmo also showed low toxicity on the Vero cells", but the dose of 78.13 ug/ml had high toxicity in Vero cells. Why were the values negative after 1250 ug/ml dose?
Also its better to provide AgNPmo treated Vero cell images in a separated Figure as an evidence to show the morphological changes in all these doses.

Response:

Thank you for this helpful comment. The Fig. 5 (now Fig. 6) legend has been revised to specify that Vero cells were cultured for 24 h prior to treatment. We have clarified in the Abstract and Results that AgNPmo shows relatively low toxicity at concentrations below 100 µg/ml (IC50 = 38 µg/ml), while higher concentrations caused marked cytotoxicity. The negative values at ≥1250 µg/ml are likely due to severe loss of metabolic activity combined with optical interference of AgNPmo with the CCK-8 assay. Unfortunately, microscopy images of treated cells were not captured during the original experiment; this has been noted as a limitation, and future work will include morphological confirmation.

Fig 6 and 7. Statistical significance on bar

Comment:
Fig 1.after the synthesis (red line), and analysis after 12 months of synthesis (blue line).: What was the necessity and significance between these two peaks

Response:
The analysis immediately after AgNPmo synthesis (red line) and after 12 months (blue line) shows the long-term stability of AgNPmo, as the absorbance wavelength peaks are similar between the two.

Comment:
Include S1 Fig. Negative control of DMSO in Fig.4 as commented above.
S1.

Response:
The Negative control has been added to Fig. 4 (now Fig. 5)

Comment:

Spectrophotometry readings and S2 Table. Spectrophotometry readings: add these peaks directly into corresponding Figure peaks. At least the major peaks can be added in Figs.

Response: The peaks have been added directly into the figure

Comment:
S3 Table: The reason for negative value (high cytotoxicity) of doses 312.5 to 5000 μg/μl should be discussed in discussion.

Response: This has been addressed in the discussion section
